# Detecting the population dynamics of an autosomal sex ratio distorter transgene in malaria vector mosquitoes

**DOI:** 10.1111/1365-2664.13702

**Published:** 2020-07-20

**Authors:** Paola Pollegioni, Ace R. North, Tania Persampieri, Alessandro Bucci, Roxana L. Minuz, David Alexander Groneberg, Tony Nolan, Philippos‐Aris Papathanos, Andrea Crisanti, Ruth Müller

**Affiliations:** ^1^ Polo d'Innovazione di Genomica Genetica e Biologia Società Consortile R.L. Terni Italy; ^2^ National Research Council Research Institute on Terrestrial Ecosystems Porano Italy; ^3^ Department of Zoology University of Oxford Oxford UK; ^4^ Institute of Occupational Medicine, Social Medicine and Environmental Medicine Goethe University Frankfurt am Main Frankfurt am Main Germany; ^5^ Department of Life Sciences Imperial College London London UK; ^6^ Liverpool School of Tropical Medicine Liverpool UK; ^7^ Department of Experimental Medicine University of Perugia Perugia Italy; ^8^ Faculty of Agriculture, Food and Environment Hebrew University of Jerusalem Jerusalem Israel; ^9^ Unit Medical Entomology Department of Biomedical Sciences Institute of Tropical Medicine Antwerp Belgium

**Keywords:** Ag(PMB)1, anopheles, dynamics, genetically modified, malaria, mosquito, populations, transgene

## Abstract

1. The development of genetically modified (GM) mosquitoes and their subsequent field release offers innovative and cost‐effective approaches to reduce mosquito‐borne diseases, such as malaria. A sex‐distorting autosomal transgene has been developed recently in G3 mosquitoes, a laboratory strain of the malaria vector *Anopheles gambiae* s.l. The transgene expresses an endonuclease called I‐PpoI during spermatogenesis, which selectively cleaves the X chromosome to result in ~95% male progeny. Following the World Health Organization guidance framework for the testing of GM mosquitoes, we assessed the dynamics of this transgene in large cages using a joint experimental modelling approach.
2. We performed a 4‐month experiment in large, indoor cages to study the population genetics of the transgene. The cages were set up to mimic a simple tropical environment with a diurnal light‐cycle, constant temperature and constant humidity. We allowed the generations to overlap to engender a stable age structure in the populations. We constructed a model to mimic the experiments, and used the experimental data to infer the key model parameters.
3. We identified two fitness costs associated with the transgene. First, transgenic adult males have reduced fertility and, second, their female progeny have reduced pupal survival rates. Our results demonstrate that the transgene is likely to disappear in <3 years under our confined conditions. Model predictions suggest this will be true over a wide range of background population sizes and transgene introduction rates.
4. *Synthesis and applications*. Our study is in line with the World Health Organization guidance recommendations in regard to the development and testing of GM mosquitoes. Since the transgenic sex ratio distorter strain (Ag(PMB)1) has been considered for genetic vector control of malaria, we recorded the dynamics of this transgene in indoor‐large cage populations and modelled its post‐release persistence under different scenarios. We provide a demonstration of the self‐limiting nature of the transgene, and identified new fitness costs that will further reduce the longevity of the transgene after its release. Finally, our study has showcased an alternative and effective statistical method for characterizing the phenotypic expression of a transgene in an insect pest population.

## Graphical abstract

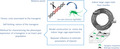

## 1 INTRODUCTION

Malaria transmitted by anopheline mosquitoes is the preeminent vector‐borne disease, infecting millions of people and accounting for hundreds of thousands of deaths every year (World Health Organization 2018). Traditional control tools based on the use of insecticides have reduced the incidence of malaria but have not resulted in its eradication (Shretta et al., 2017). Although conventional insecticides remain effective primary public health tools, current concerns about mosquito insecticide resistance and behavioural change are now driving the search for complementary vector control strategies including the development of genetically modified (GM) mosquitoes and their subsequent field release (Gabrieli, Smidler, & Catteruccia, 2014). Many different approaches have been described and classified according to the persistence or invasiveness of the genetic modifications (Burt, 2014). Self‐limiting transgenes are designed to decline in frequency and disappear from the vector population, while gene drive constructs are designed to be permanent and self‐sustaining, spreading through the initial target population without further releases (Alphey, 2013; Burt, 2014). The impact on malaria disease of both systems, self‐limiting and self‐sustaining, remains to be determined.

Self‐limiting population suppression strategies based on the release of transgenic, sterile male mosquitoes have already been proposed against the key malaria vector, *Anopheles gambiae* s.l. (Burt, 2014). However, sterilized male *Anopheles* developed via the sterile insect technique displayed severe fitness costs in terms of mating ability in comparison to their wildtype counterparts (Helinski, Parker, & Knols, 2009). Poor mating competitiveness was also observed in the case of *A*. *gambiae* s.l. Dominant Sterile Male [Ag(DSM)] strains based on Homing Endonucleases genes (HEGs; Klein, Windbichler, Deredec, Burt, & Benedict, 2012). In heterozygous Ag(DSM) males, the presence of a beta2‐tubulin promoter and consequently the expression of I‐PpoI homing endonuclease during spermatogenesis induced the selective cleavage of the ribosomal rDNA repeats and the shredding of the paternal X chromosome in sperm cells (Windbichler, Papathanos, & Crisanti, 2008). The transmission of the I‐PpoI enzyme via sperm to the eggs induced complete embryonic lethality as a consequence of the shredding of the maternal X chromosome.

Subsequently, synthetic autosomal sex ratio distorters that are fully fertile, unlike the Ag(DSM) strain, have been generated using two alternative approaches. First, Galizi et al. (2014) engineered a less stable variant of the I‐PpoI enzyme (mutation W124L) and restricted I‐PpoI activity to spermatogenesis (construct [3xP3‐DsRed]β2‐eGFP::I‐PpoI‐124L). They induced the shredding of the X chromosome during male meiosis in the testes and led to the development of the fully fertile Paternal Male Bias transgenic line [Ag(PMB)1 formerly ^gfp^124L‐2] that produced ~95% male when transgenic Ag(PMB)1 males were crossed with wildtype females only. Second, Galizi et al. (2016) described a CRISPR‐Cas9 sex distortion system targeting X‐linked ribosomal sequences. The expression of the Cas9 enzyme prevented the transmission of the X chromosome to their progeny and induced male bias ranging from 86% to 95%.

These lines combine a number of interesting features that make them appealing for future field releases of male transgenic mosquitoes, which neither transmit pathogens nor feed on humans. Large releases could deliver vector control, since the sex bias in the progeny of the released males will reduce the number of females in subsequent generations (Facchinelli et al., 2019). It is also expected that the transgene would be selected out of the population eventually, because male bias is a disadvantageous trait whenever it is common enough to skew the sex ratio of the population at large. The selection against the transgene from this factor alone is likely to decline as the transgene nears extinction, meaning it may persist as a rare allele for a prolonged period before it is eventually lost (Alphey, 2013).

It is also possible that these transgenes confer additional fitness costs in two respects. First, the small number of female progeny of transgenic males may have a reduced fitness due to I‐PpoI/CRISPR‐Cas9 damage to the X‐chromosome that is inherited. Secondly, the sperm count of transgenic males is possibly less than that of their non‐transgenic brothers, so that females mated to transgenic males may have reduced fertility. Clearly, if either of these two factors are significant, the rate of loss of the transgene from populations would be accelerated.

Although these distorters has been shown to efficiently suppress wildtype mosquito populations in small cages (17.5 × 17.5 × 17.5 cm; Galizi et al., 2014, 2016), a phased testing pathway has been recommended for the evaluation of their potential for vector control in the field (World Health Organization 2014). The phased approach includes the evaluation of the dynamics of the construct in indoor cage studies (Phase 1) followed by the evaluation of the potential for the transgene to suppress populations in physically/ecologically confined field trails (Phase 2). In particular, the critical step of Phase 1 in the testing pathway (Laboratory Population Cages) should involve (a) large cage laboratory trials with semi‐field environmental settings to predict GM mosquito competitive performance and assess the rate of population suppression, (b) GM mosquito release simulations in large indoor cages and (c) modelling effects anticipated in wild populations. As recommended by World Health Organization (2014), the prediction of transgene persistence in wildtype mosquito populations in large cages should be a component before field performance assessments. As already demonstrated for *Wolbachia* drive systems developed in *Aedes aegypti* (Hoffmann et al., 2011; Walker et al., 2011), simulating the natural environment within contained settings provides the opportunity to incorporate more realistic and repeatable observations of behaviour, and assists model parameterization (Mumford, Leach, Benedict, Facchinelli, & Quinlan, 2018).

Here, we observed the dynamics of the [3xP3‐DsRed]β2‐eGFP::I‐PpoI‐124L transgene carried by Ag(PMB)1 strain in indoor large cage populations over a 4‐month period. In addition, we performed life‐history assays in similar conditions to characterize the development and survival of mosquitoes depending on their age, sex and genotype (transgenic or not). The cages allowed stable age structured populations of *A. gambiae* s.l. to be monitored in conditions reproducing a simple tropical environment, with a diurnal light cycle and constant temperature and humidity. We used Bayesian inference to combine the ensuing data with a stochastic simulation model to estimate parameters of interest. In particular, we inferred fitness costs associated with the transgene alongside the extent of paternal male bias. Finally, we used the best fitting model to predict the extinction time of the transgene in different scenarios with respect to population size and the initial inoculation of transgenic mosquitoes.

## 2 MATERIALS AND METHODS

### 2.1 Wildtype and Ag(PMB)1 strains

The Ag(PMB)1 transgenic line was developed from the G3 wildtype colony derived from the Gambia (stock number MRA‐112), which has been maintained in the laboratory for 43 years (stored at Malaria Research and Reference Reagent Resource Center, 2017) and is recognized as a hybrid stock between *A. gambiae* and *Anopheles coluzzii* (*A. gambiae* s.l.) (https://www.vectorbase.org/organisms/anopheles‐coluzzii/g3). Since the Ag(PMB)1 strain has been maintained in the laboratory by continuously crossing transgenic females to G3 males, we expect the two lines to differ only in terms of the presence or absence of the transgene. The non‐transgenic G3 line is thus an appropriate comparator for identifying transgenic effects, and we henceforth refer to G3 mosquitoes as ‘wildtype’ (a full description of the two strains is in the Appendix S1). The transgenic mosquitoes were identified by screening for the expression of the 3xP3‐DsRed fluorescent marker.

### 2.2 Overlapping generation large cage experiment

**FIGURE 1:**
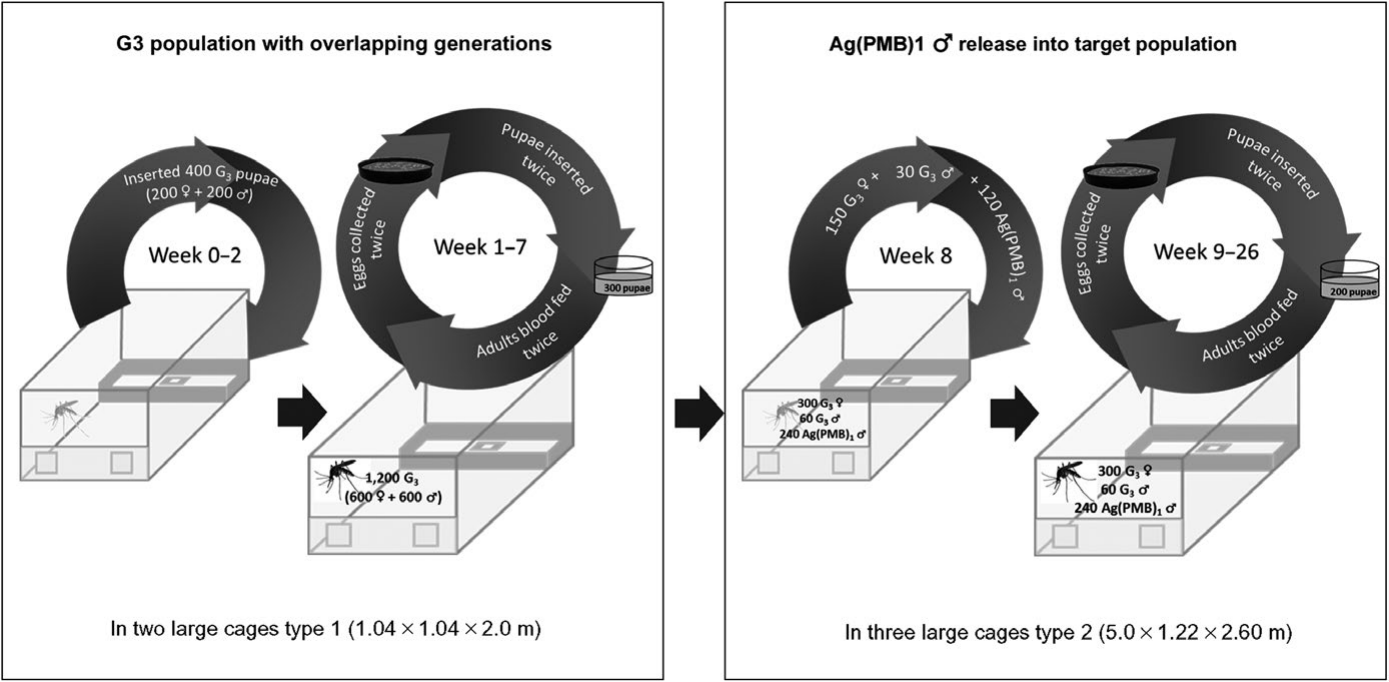
Testing the frequencies of the Ag(PMB)1 adults in age‐structured, generation‐overlapping wildtype populations. First, two age‐structured, generation‐overlapping populations using the G3 strain were stabilized in two large cages type 1 (1.04 × 1.04 × 2.0 m; week 1–7). Second, age‐structured, generation‐overlapping populations were transferred to three large cages type 2 (week 8) and 2‐day‐old heterozygous Ag(PMB)1 males were introduced into G3 populations (week 9–26)

Our experiment to monitor transgene dynamics in generation‐overlapping populations was divided into two stages, as described below (Figure 1). In both stages, the temperature was kept at 27°C (±1°C) and relative humidity at 75% (±10%). As described by Facchinelli et al. (2019), the cages were arranged with swarming stimuli and food sources. In order to promote the swarming process of male mosquitoes in each cage, dawn lasted for 30 min from dark to full light, and full light lasted for 11 hr 30 min. Sunset lasted for 1 hr 30 min, overlapping with 60 min of twilight provided by horizon lights.

### 2.3 Establishment of stable age‐distributed wildtype populations

First, we established a stable age distribution of wildtype adult mosquitoes by maintaining wildtype populations in two large (1.04 × 1.04 × 2.0 m) cages, that we will refer to as ‘type 1’, for 7 weeks. Each population was established by adding 400 G3 2‐day‐old adults weekly from week 0 to week 2 to obtain initial populations of 1,200 mosquitoes (600 males and 600 females; Figure 1). Adult females were fed twice a week (Mondays and Thursdays) with defibrinated and heparinized sterile cow blood (Allevamento Blood di Fiastra Maddalena) using a Hemotek membrane feeder (DiscoveryWorkshops), and on each occasion, eggs were collected the following day and counted. We reared sufficiently many eggs from each twice‐weekly batch to return 300 pupae to the cages from each cohort, to maintain the populations. This protocol resulted in two cage populations of approximately 1,500 adult mosquitoes at the end of week 7, with age distributions that we could reasonably assume to be stable.

### 2.4 Large cage populations comprising both wildtype and Ag(PMB)1 mosquitoes

In week 8, we transferred 300 females and 60 males from the two stable age wildtype populations into each of three large (5.0 × 1.22 × 2.6 m) cages that we will refer to as type 2, using a backpack aspirator to collect the mosquitoes. In this way, each wildtype population comprised a mixture of virgin, unmated and already mated females and males. We then added 240 virgin 2‐day‐old Ag(PMB)1 heterozygous males, so that each cage contained a total of 600 mosquitoes and the transgene allelic proportion was 20% (Figure 1). From the establishment of the target population until the first progeny release into the type 2 cages (on day 14), the expected adult mortality of mosquitoes in these cages (Table S1 in the Appendix S2) was compensated by a second introduction of 300 2‐day‐old virgin adult mosquitoes: 150 G3 females, 30 G3 males and 120 Ag(PMB)1 males (Figure 1).

Subsequently we recorded the total number of eggs, hatching rate, larval survival, pupal mortality rates and the ratio of non‐transgenic to transgenic mosquitoes among the progeny in the three replicate populations over the course of 4 months. As in the type 1 cages, females were blood‐fed and eggs collected twice a week. All eggs from each oviposition were placed in a single tray with 300 ml of deionized water for hatching and 5 ml of 2% w/v larval diet. The hatching rate was calculated after 48 hr according to the protocol in Lehmann et al. (2006). Afterwards, 500 first‐instar larvae were randomly selected from each cage, allotted to two trays and maintained using a standard rearing protocol until they reached the fourth instar. For each tray, the number of the initial 250 larvae that successfully reached the pupal stage was counted, providing data on the larval survival. At the pupal stage, a subset of 200 individuals was randomly selected and screened for fluorescence (3xP3‐DsRed marker) twice per week using an Evos F1 microscope (Life Technologies Italia). The selected non‐transgenic and transgenic pupae were sexed by observing their genital morphology and reintroduced into the cage from which they originated. Pupal mortality that occurred between placing pupae to large cages and adult eclosion was recorded.

After 4 months of monitoring, we checked for variations in terms of number of eggs, hatching rate, larval survival and pupal mortality as functions of cage and time, using a GLM with binomially or negative binomially distributed errors and a logit link function (Wald statistics, pit.trap resampling with 250 iterations), as implemented in the r‐mvabund package. Thereafter, a post‐hoc Tukey's HSD test was performed to investigate differences between cages and time points, using r‐lsmeans and r‐multcomp packages. A mixed effects model analysis was performed to determine whether overall observed Ag(PMB)1 frequencies statistically departed between the three large cages, using the r *‐*package nlme (Facchinelli et al., 2019; Hoffmann et al., 2011). All tests were computed using R v3.4.0 statistical programming language (R Core Team, 2017).

### 2.5 Additional survival measurements of wildtype and Ag(PMB)1 for model parametrization

In addition to the overlapping generation experiments, we also measured traits relating to survival and longevity that would not be easy to infer from the cage experiment alone. Specifically, we measured (a) the adult life span distributions for each sex and genotype in type 2 cages, and (b) the larval and pupal mortality rates for each sex, genotype and parentage (for methods see Appendix S2). In the latter case, we were particularly interested to examine whether female progeny from transgenic males had different pupal survival rates to other females. Note that while these females may or may not be themselves transgenic, their paternally inherited X‐chromosome will have been exposed to the I‐PpoI enzyme and thus may be damaged. Finally, we collected available life‐history data of Ag(PMB)1 from previous studies (Table S1 in Appendix S2).

For testing statistical differences between Ag(PMB)1 and wildtype strains in terms of larval survival and pupal mortalities, we used a generalized linear model (GLM) with binomially distributed errors and a logit link function as previously described. Adult longevity functions for Ag(PMB)1 and wildtype strains were estimated using the non‐parametric Kaplan–Meier estimator based on right censored observations and implemented in the r‐survival package v2.37‐4. We tested putative variation in the 50% adult longevity (days) among Ag(PMB)1 and wildtype adults in type 2 cages and two blocks of replicates corresponding to two different times of data recording using the two‐way analysis of variance. We fitted Weibull distributions to our survival data for each sex and genotype.

### 2.6 The simulation model and parameter inference

To investigate transgene fitness effects from these data, we constructed a stochastic individual based simulation model of the cage population experiments. The model is fully described in the Appendix S3, and was implemented in ‘C++’ (Appendix S4).

We set our model assuming stable age distribution of initial G3 wildtype adults in the target populations and the life‐history traits reported in the Table S1 of the Appendix S2. We inferred key parameters of the model by comparing simulations to the experimental data using a Monte Carlo algorithm based on the method of ‘approximate Bayesian computation’ (ABC) (Csilléry, Blum, Gaggiotti, & François, 2010), as follows. First, a parameter vector was drawn at random from a prior distribution. Second, simulations were run with this parameter vector. Third, the simulated data were scored for their closeness to the cage‐observed data according to a number of distance measures. We then repeated these three steps many times (>10^5^) to obtain a set of parameter vectors and associated distance values. Finally, we selected the best‐fitting parameter vectors by retaining those for which each distance score was in the lowest 0.3014 quantile. This value was determined so as to return 200 posterior points from 200,000 samples from the prior distribution. We judged this to be a satisfactory balance between the number of posterior points (which increases with the filtering quantile) and the concentration of posterior density (which reduces with the quantile; Csilléry et al., 2010). The retained parameter vectors formed our estimated posterior distribution. The specific distance measures and the prior distribution that we used are described in Appendix S5.

By this method, we estimated the following five model parameters. (a) The fertility cost to females from mating with an Ag(PMB)1 male (*ϕ*). (b) The fertility cost to females from carrying an X‐chromosome produced by an Ag(PMB)1 male ancestor (*ψ*). (c) The extent of male bias in the gametes produced by Ag(PMB)1 males (*b*). (d) The probability that a given female successfully blood feeds and oviposits after a blood meal (*p*_Lay_). Finally, (e), the average number of eggs laid by those females that do oviposit after a blood meal (*θ*).

### 2.7 Using the posterior distribution

We used the posterior‐informed model to investigate the persistence of the transgene in wildtype populations. We simulated four initial transgene frequencies (1%, 5%, 10% and 20%), and in each simulation we randomized the twice‐weekly egg‐restocking rate (in the range 100–2,000) in order to explore the importance of population size. The initial transgene frequency was set by assuming half the mosquitoes introduced to the type 2 cages were G3 females and adjusting the ratio of Ag(PMB)1 heterozygous males to G3 males to achieve the desired overall frequency. We ran each simulation until a time when there were no transgenic larvae.

## 3 RESULTS

### 3.1 Overlapping generation large cage experiment

**FIGURE 2:**
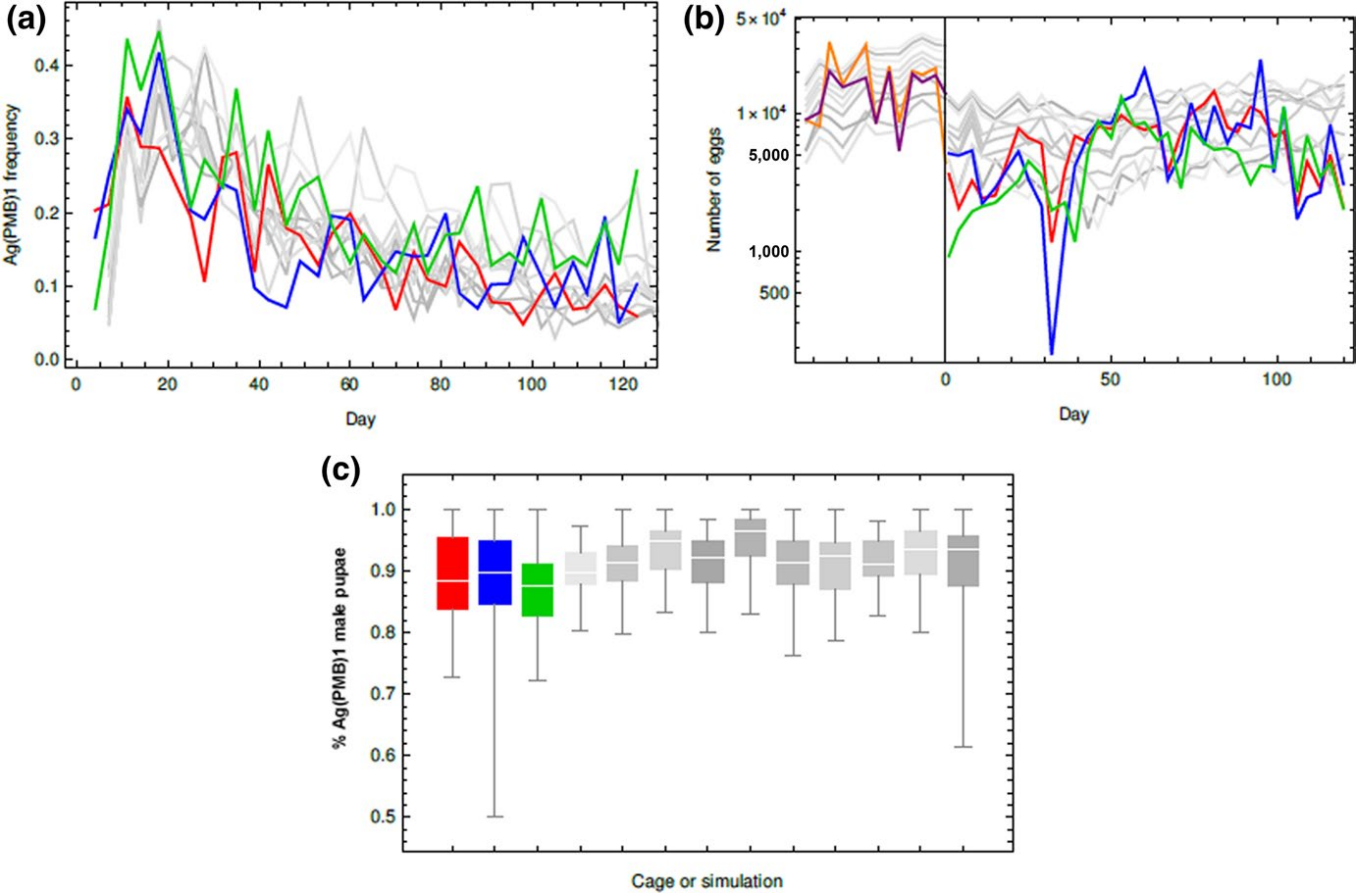
Cage population dynamics in terms of (a) the total proportion of transgenic Ag(PMB)1 pupae, (b) the number of eggs and (c) the male proportion among transgenic pupae through the course of the experiment. Experimental outcomes are shown in red, blue and green lines for the cage A, cage B and cage C, respectively, while the grey lines/distributions show 10 model simulations using the posterior‐informed model. The total egg production in the two type 1 cages in the pre‐transgenic release phase is shown in orange (cage 1) and violet (cage 2)

In the two type 1 cages used in the first part of the experiment to establish stable age distributed wildtype populations, the total number of eggs laid varied substantially from week to week (in the ranges [4,396–33,395] and [5,385–20,573]). High population densities were observed in both replicates by week 2 (Figure 2).

The egg laying rate was again highly variable in the remaining part of the experiment, after the populations were transferred into the type 2 cages and the Ag(PMB)1 males were introduced (the egg number ranges were [1,161–14,714], [175–25,045] and [919–13,227] in the three cages; Figure 2; Table 1). It was expected that egg number would reduce in the type 2 cages because we reduced the restocking rate in this part of the experiment (see Section 2). A significant difference in egg production was detected between cages A and B and cage C (Wald test = 2.75, *df* = 2, *p* < 0.05) with females from cage C producing fewer eggs. In addition, the mean number of eggs laid every week increased steadily in a short temporal window from day 36 to day 50, and was positively correlated with a progressive increase in hatching rate (*R*‐Spearman = 46.85%, *p* < 0.01) and in the frequency of wildtype males among the progeny (*R*‐Spearman = 61.78%, *p* < 0.01; Figure S1 in Appendix S6). Similarly, the mean hatching rates of cage C were slightly lower than cages A and B and comparable to the expected value. Similar to the total number of eggs, hatching rates increased from day 36 to day 50 (Table 1; Figure S1 in Appendix S6). Although we observed significant fluctuations through time, larval and pupal mortality did not vary between the type 2 cages (Table 1; Figure S2 in Appendix S6), and never exceeded the expected mortality ranges of wildtype and Ag(PMB)1 mosquito strains (Table S1 in Appendix S2).

**TABLE 1:** Average and time‐dependent life‐history traits recorded over 120 days. Mean (±*SE*) total number of eggs, hatching rate, larval mortality, total pupal mortality, male pupal mortality and female pupal mortality of non‐transgenic and Ag(PMB)1 mosquitoes recorded after seeding each large cage with 20% transgene frequency (large cage A–C). Statistical differences between cages (Cage effect) and over the experimental course of 4 months (Time effect) were tested using binomial generalized linear model (GLM)

Generalized linear model statistics							
		Wald test (cage effect)	*df*	*p* value	Wald test (time effect)	*df*	*p* value
Total number of eggs							
Cage A	6,580 ± 550	2.75	2	<0.05	16.36	32	<0.01
Cage B	7,183 ± 932						
Cage C	4,963 ± 514*						
Hatching rate							
Cage A	0.896 ± 0.006	7.29	2	<0.01	12.50	33	<0.01
Cage B	0.889 ± 0.005						
Cage C	0.858 ± 0.009*						
Larval mortality							
Cage A	0.099 ± 0.012	2.67	2	0.05	30.16	33	<0.01
Cage B	0.107 ± 0.012						
Cage C	0.107 ± 0.014						
Total pupal mortality							
Cage A	0.081 ± 0.005	1.24	2	0.05	17.28	33	<0.01
Cage B	0.086 ± 0.009						
Cage C	0.083 ± 0.009						
♂ Pupal mortality							
Not‐transgenic ♂							
Cage A	0.081 ± 0.006	0.328	2	0.94	12.13	33	<0.01
Cage B	0.083 ± 0.010						
Cage C	0.086 ± 0.010						
Ag(PMB)1 ♂							
Cage A	0.077 ± 0.012	3.649	2	<0.01	12.93	33	<0.01
Cage B	0.120 ± 0.024						
Cage C	0.084 ± 0.013						
♀ Pupal mortality							
Not‐transgenic ♀							
Cage A	0.083 ± 0.007	0.53	2	0.598	8.93	33	<0.01
Cage B	0.073 ± 0.007						
Cage C	0.071 ± 0.008						
Ag(PMB)1 ♀							
Cage A	0.1436 ± 0.041	2.39	2	0.058	4.14	33	0.443
Cage B	0.2209 ± 0.047						
Cage C	0.1213 ± 0.030						

Notes

*Significant difference at *p* < 0.05 according to the post‐hoc Tukey's HSD test after providing significance within GLM.

The Maximum Likelihood parameterization of the mixed‐effects models indicated that Cage A and Cage B did not differ from each other in the proportion of Ag(PMB)1 individuals observed over four months (L.Ratio_(6,4)_ = 1.043, *p* = 0.59), yet both departed from Cage C which tended to have a higher Ag(PMB)1 proportion (A: L.Ratio_(6,4)_ = 13.56, *p* = 0.001; B: L.Ratio_(6,4)_ = 9.018, *p* = 0.011). However, the overall transgene frequency trends were similar in all three cages (Figure 2). Initially, the proportion of transgenic mosquitoes rose to a peak of 35%–44%, as wildtype females mated with the introduced Ag(PMB)1 and began producing offspring. This phase lasted between 10 and 20 days depending on the cage. Note that the wildtype female population was approximately stable age‐distributed when we introduced the Ag(PMB)1 males, and probably most of the females were already mated at this time. We thus expect that the initial increase of the transgene was slower and reduced in extent than it would have been if, instead, we had introduced all wildtype females as pupae alongside the Ag(PMB)1 male pupae.

Thereafter, the transgene gradually declined in proportion, though with considerable variability. In the last round of observations, the frequency of Ag(PMB)1 mosquitoes was between 6% and 26% depending on cage (Figure 2). The sex ratio among transgenic pupae observed among progeny for four months ranged from 50% to 100% male across all (twice weekly) samples and all three cages, with an overall mean value of 87.7% male. Note that this is not a direct measurement of the sex bias caused by I‐PpoI expression in transgenic males, since some transgenic pupae may be the progeny of transgenic females. However, these data are important to the inference of paternal male bias.

### 3.2 Survival measurements

No significant differences in larval mortality (Wald test = 2.08, *df* = 1, *p* = 0.06), total pupal mortality (Wald test = 2.05, *df* = 1, *p* = 0.05), male pupal mortality (Wald test = 1.88, *df* = 1, *p* = 0.08) and female pupal mortality (Wald test = 1.02, *df* = 1, *p* = 0.29) were detected between transgenic Ag(PMB)1 and wildtype individuals. However, pupal mortality was significantly higher in females (whether transgenic or not) that had inherited an X‐chromosome from an Ag(PMB)1 father than in all other pupae (Wald test_transgenic_ = 2.11, *df* = 1, *p* < 0.05; Wald test_non‐transgenic_ = 2.07, *df* = 1, *p* < 0.05; Table S1 in Appendix S2).

No significant differences in adult survival between Ag(PMB)1 and wildtype were detected in type 2 cages (*F* = 5.08, *df* = 1, *p* = 0.05), with no block effect between replicates (*F* = 0.33, *df* = 1, *p* = 0.58). However, sex‐specific difference in adult longevity were detected in both strains with females living longer than males (for a full description see Appendix S2). We therefore fitted the Weibull survival curves separately for each sex after pooling the genotype‐specific data, to obtain shape (*k*) and scale (*λ*) parameters: $k_{\text{male}}=1.8,\;\lambda_{\text{male}}=16.3,\;k_{\text{female}}=2.2$, and $\lambda_{\text{female}}=20.2$.

### 3.3 Parameter inference from the cage data

**FIGURE 3:**
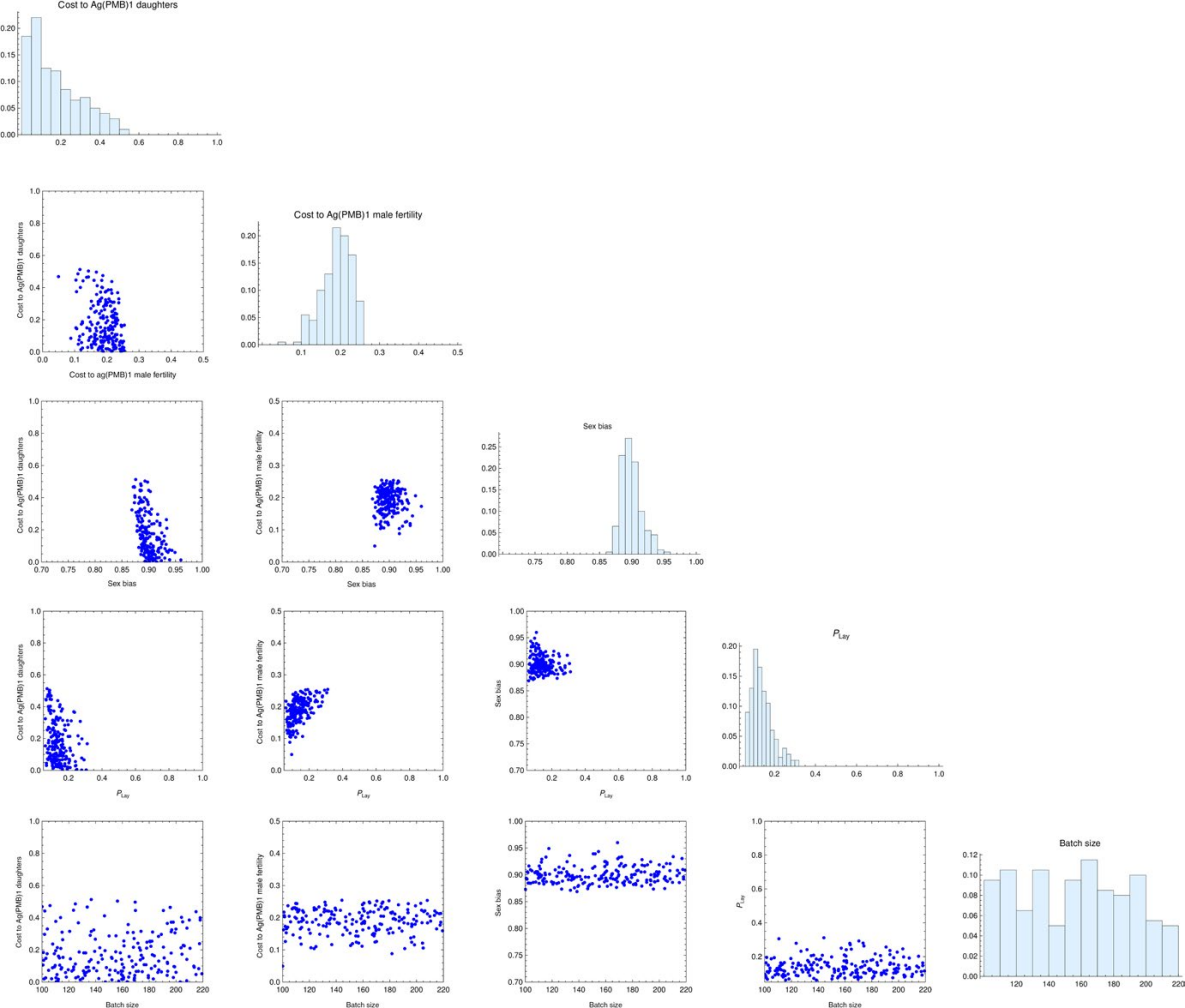
Inferring posterior probability of five model parameters: cost to Ag(PMB)1 daughters (*ψ*) cost to Ag(PMB)1 male fertility (*ϕ*), the extent of paternal male bias (b), the probability that a given female successfully blood feeds and oviposits after a blood meal (*p*_Lap_) and the batch size of eggs laid by those females that do oviposit after a blood meal (*θ*). The plotted points and associated histograms show the inferred posterior distribution of the 200 best fitting parameter combinations

The posterior parameter distribution analysis is shown in Figure 3. There is clear evidence that females, transgenic and non‐transgenic, mated to transgenic males have around 20% lower egg production than females mated to wildtype males. There is less support for reduced fertility among adult females carrying an X‐chromosome from an Ag(PMB)1 male, though our analysis cannot rule out this possibility. These females are rare, due to both Ag(PMB)1 male biasing and also because they have lower pupal survival (Table S1 in Appendix S2). The dynamics of the transgene are therefore insensitive to adult female fertility costs of the affected X‐chromosome, hence it is unsurprising that our analysis is unable to accurately infer this parameter.

The male bias in viable gametes produced by Ag(PMB)1 males is estimated to be somewhat lower in the overlapping generation cage experiment, at around 88%–92% male offspring (mean 90.0%), than has been estimated in small cage crossing experiments (94.6% ± 0.9%). The results also suggested that only about 15% of females oviposit on each egg collection day (mean estimate 14.5%). It is not possible to infer typical egg batch size from these data, which may be because the experimental data showed considerable variations in egg number both between the different cages and between cohorts in each individual cage.

Simulations of the cage dynamics with parameters drawn at random from the posterior distribution correspond closely to the observed transgene frequency dynamics and transgene paternal male bias, indicating that the fitted model captures much of the biology of the transgene kinetics (Figure 2). The simulations perform less well in replicating the variability in egg number seen in the cages, suggesting the model does not successfully incorporate all the sources of this variation.

### 3.4 Predicted Ag(PMB)1 persistence time

**FIGURE 4:**
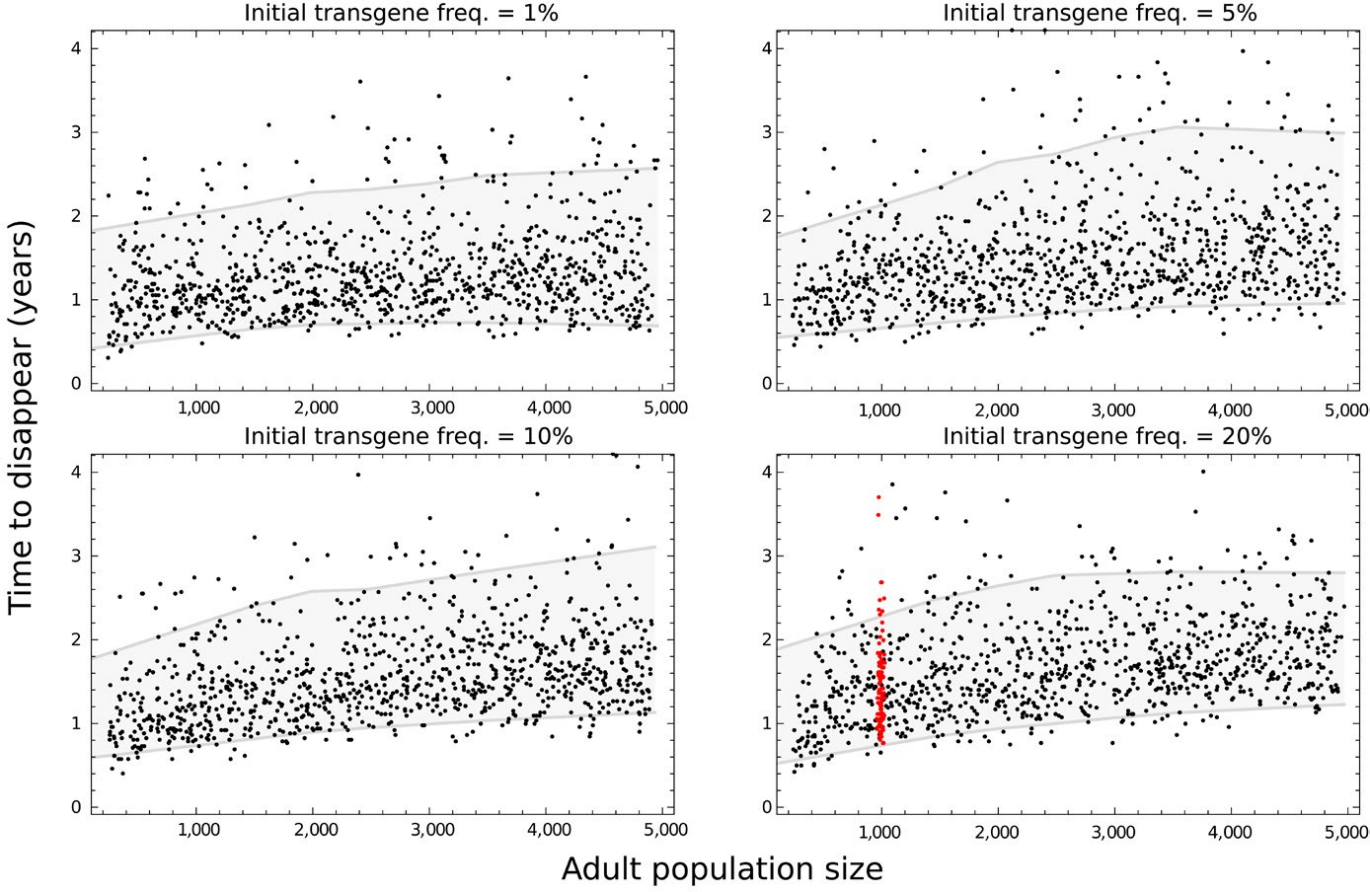
Predictions of transgene longevity in the population over a range of population sizes and initial transgene proportions. Each point plots the time to disappearance of the transgene in a simulation of the posterior‐informed model, against the average adult population size in that simulation (post release). The population size was varied among simulations by varying the number of pupae added back into the population from each twice‐weekly cohort of eggs. The introduced transgenic frequency in the cage experiment was 20%, and for this panel the red points indicate that the model simulated the restocking rate that was used in the population experiment. The grey region shows the 90% central quantile of the points

Our simulations with the posterior‐informed model suggest that both population size and the initial frequency of transgenic mosquitoes will have a modest influence on the mean persistence of Ag(PMB)1 in a population, which will be generally longer in large populations and following large initial transgene releases (Figure 4). The effects of both these factors are small, however, in comparison with the considerable variability in persistence time for any particular scenario.

For the population size and introduction frequency that were used in our type 2 cage experiment, the mean disappearance time of Ag(PMB)1 is predicted to be 529 days with 95% of simulations falling between 308 and 896 days (Figure 4). However, the duration of persistence is variable because the transgene is a rare allele for much of the time that it is extant in the population. The duration of this phase depends on the inherently stochastic process of genetic drift, hence the overall variability. The transgenic release size is relatively unimportant because it mainly affects the duration of an initial phase of decline, in which the transgene drops from its introduction frequency to rarity. Population size is somewhat more important because larger populations are less affected by genetic drift, so that random fluctuations in allele frequency are reduced and slower to cause extinction of the transgene.

## 4 DISCUSSION

In this study, we observed the dynamics of the transgene carried by Ag(PMB)1 mosquitoes in indoor large cage wildtype populations over a 4‐month period. These experiments are in line with World Health Organization (2014) guidance recommendations in regard to the development and testing of self‐limiting GM mosquito technologies.

A previous study investigated the impact of multiple Ag(PMB)1 releases into indoor large cage wildtype populations (Facchinelli et al., 2019). These releases led to male‐biased adult populations which were smaller than control populations that did not contain Ag(PMB)1 mosquitoes. An associated stochastic model predicted these trends, though with somewhat larger effect sizes and lower variability. In the current study, we relaxed the assumptions of the model used by Facchinelli et al. (2019) in two respects. We incorporated possible transgene fitness costs. Our model allows the possibility of Ag(PMB)1 males having reduced fertility, and also the possibility that X‐chromosomes produced by Ag(PMB)1 males confer lower fitness in females. We also incorporated heterogeneous egg‐laying among adult females, by assuming that only a fraction of females successfully bloodfeed and oviposit at each opportunity. We have shown that both these factors are important to fully understanding the transgene dynamics. The preclusion of fitness costs in the model of Facchinelli et al. (2019) is therefore the probable reason for its overestimation of effect sizes, while the preclusion of egg laying variation is the probable reason for its underestimation of variability in the transgene dynamics.

The detection of fitness costs associated with the transgene carried by the Ag(PMB)1 strain is particularly important to predicting how long it might persist in populations where it is introduced. Though the fitness costs that we detected are not substantial, our posterior‐informed model predicts that the transgene invariably disappears within a few years of its introduction, regardless of its initial frequency and over a wide range of population sizes. These persistence times would be considerably longer if there were no fitness costs, though extinction is always predicted to occur eventually because the male‐biasing trait is disadvantageous when common. The heterogeneity of egg laying is responsible for a large part of the variation in predicted persistence times among simulations. Our model gave the best fit to the data when only ~15% of females lay eggs at each opportunity, which results in considerable genetic drift acting on the transgene. This and other sources of stochasticity mean that there is inherent unpredictability to the precise persistence time of the transgene in a population. However, the use of stochastic simulations can be effective in predicting lower and upper bounds of persistence.

Our data indicated a somewhat lower level of paternal male biasing caused by the expression of the I‐PpoI endonuclease during spermatogenesis in Ag(PMB)1 male mosquitoes than has been found previously. We estimated that the offspring from Ag(PMB)1 males are ~90% male, whereas Galizi et al. (2014) observed ~95% male offspring from small cage crossing experiments. The reasons for this difference are unclear, and further studies would be useful.

The inference of mosquito parameters from population level time series data has become increasingly prevalent in recent years (e.g. Hancock et al., 2016; Ng'habi et al., 2018; Valerio et al., 2016), yet an alternative approach is to measure the parameters of interest in separate experiments (e.g. Kyrou et al., 2018; McArthur, Meredith, & Eggleston, 2014). Here, we have combined the two methods by measuring survival parameters in addition to inference from a population level experiment. We chose this approach because the two methods have distinct strengths. Arguably, parameter‐specific experiments give information on the specified parameters that is more precise, because confounding factors are controlled. Furthermore, these experiments can enable a mechanistic understanding of phenotypic effects, which is not usually possible from population level data alone. For example, our population‐level inference indicated that Ag(PMB)1 males have reduced fertility over their wildtype counterparts, yet it is difficult to discern whether this is due to a reduced sperm count, to less viable sperm, or to reduced mating competitiveness. Data from parameter‐specific experiments are generally straightforward to interpret, while the reliability of parameter inference from population trends depends on the ability of the model to capture the true biology of the population. The close correspondence between simulations of our fitted model and the transgene frequency data gives us confidence that the model does capture the important features of the biological system, though we acknowledge the model understates the egg‐laying variability that was observed. We could construct models with greater complexity to help resolve this issue, though model complexity comes with a cost to parameter inference while also increasing the risk of overfitting.

Despite these challenges, inference from population level data has important advantages over separate experiments. The method does not require the experimenters to know in advance which parameters are important. In the current study, for example, separate experiments might have neglected to investigate the heterogeneity of egg laying among females, yet our analysis identified the importance of this factor to the variability of transgene persistence. Separate experiments might miss transgene effects if, for example, a trait is not sufficiently affected for the null hypothesis to be rejected. Separate experiments might miss interactions among traits, yet population level trends will subsume the effects of important interactions. Finally, the analysis of population level trends allows the importance of model parameters to be assessed alongside their estimation. For example, our difficulty in discerning whether the female descendants of Ag(PMB)1 males have lower fertility is, in part, because these females are rare in the population. Such a fertility cost is therefore not greatly consequential to the transgene dynamics.

We chose to measure survival rates separately from the population experiment because these parameters would be difficult to infer from the population data alone, while their uncertainty would confound inference of the other parameters of interest. Our study thus demonstrates that the two general methods of parameter estimation are complementary. Further parameter‐specific experiments will help to corroborate the inferences from population level data. For example, our finding that Ag(PMB)1 males have reduced fertility could be further investigated with experiments aimed at discerning the mechanism of this effect.

We are aware that our experiments were performed under relatively simple and uniform laboratory conditions, which only partially reflect field conditions. Our modelling predictions should thus be considered as a first step to exploring the persistence of the transgene carried by Ag(PMB)1 strain in different types of population. Before a field release programme, it will be important to develop models based on the specific geography of the release site, that are informed as far as possible from local field data. Such modelling will help evaluate the significance of factors that are not present in our indoor cage study, including natural predators, climatic fluctuations and the presence of other vector control measures.

## 5 CONCLUSIONS

In this study, we have investigated an important transgenic strain of a key malaria vector. The transgene is interesting because it has the potential to suppress vector populations if released in large numbers, though the suppressive effects are expected to short‐lived. The ephemerality makes the transgene a less powerful genetic technology than a gene drive (Burt, 2014), yet also one which may be approved for field testing sooner. Since the transgenic strain (Ag(PMB)1) has been considered for genetic vector control of malaria, our results are fundamentally important for determining expectations on the persistence of the transgene post‐release. Our results provide a demonstration of the self‐limiting nature of the transgene, and indicate that longevity will be further reduced by fitness costs that were not previously identified. Finally, our study has showcased an effective method for characterizing the phenotypic expression of a transgene in an insect pest population, and we hope that other researchers may find this instructive.

## AUTHORS' CONTRIBUTIONS

P.P., A.R.N., T.N., P.‐A.P., A.C. and R.M. conceived the ideas and designed methodology; P.P., A.R.N., T.P., A.B. and R.L.M. collected the data; P.P., A.R.N., T.P., D.A.G. and R.M. analysed the data; P.P., A.R.N., A.C. and R.M. led the writing of the manuscript. All authors contributed critically to the drafts and gave final approval for publication.

## Supporting information

Appendix S1

Appendix S2

Appendix S3

Appendix S4

Appendix S5

Appendix S6

## Data Availability

Data available from the Dryad Digital Repository: https://doi.org/10.5061/dryad.jdfn2z382 (Pollegioni et al., 2020).
