# Supplementary material for: Detecting the population dynamics of an autosomal sex ratio distorter transgene in malaria vector mosquitoes
Source: J Appl Ecol. 2020 Jul 20;57(10):2086–96. doi: 10.1111/1365-2664.13702 (PMC7594489; doi:10.1111/1365-2664.13702)
Supplement: Supplementary file 1 — Appendix S1 [file JPE-57-2086-s001.pdf]

Appendix S1. Description of comparator wild-type and transgenic Ag(PMB)1 strains

Mosquito strains

G3 is a wild-type colony (stock number MRA-112) which has been maintained in the laboratory for 43 years (Malaria Research and Reference Reagent Resource Center 2017). This strain derives from a region in West Africa (the Gambia) where two incipient species, *An. gambiae* and *An. coluzzii,* coexist. G3 strain is assumed to be an inter-specific hybrid between *An. gambiae* and *An. coluzzii,* but no complete genome sequence data are still published to confirm this assumption. However, as VectorBase reported "On the basis of both inversion polymorphisms and rDNA characteristics, this colony has been suggested to represent a mix of *Anopheles gambiae* and *Anopheles coluzzii*. Since this colony has been distributed broadly among different institutions without any specific molecular or inversion efforts at quality control, it should probably simply be recognized as a hybrid stock (*An. gambiae s.l.*)" (https://www.vectorbase.org/organisms/anopheles-coluzzii/g3).

Ag(PMB)1 transgenic line was developed from the G3 strain and classified as a sex ratio distorter - autosomal X-shredder based on I-PopI homing endonuclease (Galizi et al., 2014). This strain is marked with the fluorescent marker *3XP3DsRed* and green fluorescence protein (GFP), the latter of which was fused to I-PpoI. Both GFP and the I-PpoI enzyme were expressed in sperm. Due to the sex ratio distortion conferred by the transgene, Ag(PMB)1 strain has been maintained in the laboratory by continuously backcrossing Ag(PMB)1 females to G3 males. The transgenic mosquitoes used for these experiments were screened for the expression of the *3xP3 DsRed* fluorescent marker using a Complex Object Parametric Analyzer and Sorter (COPAS, Union Biometrica, Boston, USA). This backcrossing procedure promoted a high genetic similarity between transgenic individuals and wild-type counterparts, except for the transgene itself (Galizi et al., 2014). Life-history parameters of wild-type and transgenic Ag(PMB)1 mosquito strains were

collected from literature or experiments (please see subchapter of the Material and Methods *Additional survival measurements of wild-type and Ag(PMB)1 for model parametrization*).

Mosquito strains were maintained and reared in a climate-controlled room at a temperature of 27 ± 1°C and 75% ± 10% relative humidity, with a 12:12h light and dark regime. The mosquitoes were reared as described in Valerio et al. (2016) with the following modifications. Adult mosquitoes were carried in plastic cages of 17.5 × 17.5 × 17.5 cm and fed *ad libitum* with a 10% sucrose solution with 0.1% of methylparaben (Benedict, Hood-Nowotny, Howell, & Wilkins 2008). Females were blood fed weekly with defibrinated and heparinized sterile cow blood (Allevamento Blood di Fiastra Maddalena, Teramo, Italy) using a Hemotek membrane feeder (DiscoveryWorkshops, Accrington, UK). Eggs were collected and placed in a single tray with 350 mL of deionized water for hatching and provided 5 mL of 2% w/v larval diet. Subsequently, larvae were allotted to three trays (each containing about 250 larvae) and maintained according to the standard rearing protocol based on Damien's larval diet as the food source (Damiens, Benedict, Wille, & Gilles 2012). Pupae were collected and placed in small crystallization dish glass inside a fresh adult cage for emergence.

References

Benedict, M., Hood-Nowotny, R., Howell, P., Wilkins, E. (2008). Methylparaben in *Anopheles gambiae* s.l. sugar meals increases longevity and malaria oocyst abundance but is not a preferred diet. Journal of Insect Physiology, 55, 197-204. doi:10.1016/j.jinsphys.2008.11.003

Damiens, D., Benedict, M.Q., Wille, M., Gilles, J.R.L. (2012). An inexpensive and effective larval diet for *Anopheles arabiensis* (Diptera: Culicidae): eat like a horse, a bird, or a fish? Journal of Medical Entomology, 49(5), 1001–1111.

Galizi, R., Doyle, L.A., Menichelli, M., Bernardini, F., Deredec, A., Burt, A.,…Crisanti A. (2014). Synthetic sex ratio distortion system for the control of the human malaria mosquito. Nature Communications, 5, 3977. doi:10.1038/ncomms4977

Valerio, L., North, A., Collins, M.C., Mumford, J.D., Facchinelli, L., Spaccapelo, R., Benedict, MQ. (2016). Comparison of model predictions and laboratory observations of transgene frequencies in continuously-breeding mosquito populations. Insects, 7, E47. doi:10.3390/insects7040047.
