# Supplementary material for: Detecting the population dynamics of an autosomal sex ratio distorter transgene in malaria vector mosquitoes
Source: J Appl Ecol. 2020 Jul 20;57(10):2086–96. doi: 10.1111/1365-2664.13702 (PMC7594489; doi:10.1111/1365-2664.13702)
Supplement: Supplementary file 2 — Appendix S2 [file JPE-57-2086-s002.pdf]

Appendix S2. Computing larval mortality, pupal mortality and type 2-cage (5.0 × 1.22 × 2.60 m) adult longevity of wild-type and Ag(PMB)1 strain.

Methods

Larval mortality and pupal mortality

First-instar larvae were obtained by crossing 150 Ag(PMB)1 females × 150 G3 males in a small cage (17.5 × 17.5 × 17.5 cm) and separated by screening for fluorescence (3XP3DsRed marker, COPAS). Two thousand Ag(PMB)1 or wild-type non-transgenic sibling first-instar larvae were transferred into eight trays, each containing 250 larvae, and maintained using our standard rearing protocol until they reached 4th instars. For each of the eight trays, the number of larvae successfully reaching pupal stages was counted, providing the average percentage of larval survival for Ag(PMB)1 and wild-type non-transgenic individuals. In each tray, all pupae were collected and sexed. Pupal mortality that occurred between placing pupae in large cages and adult eclosion was recorded in eight trays for wild-type non-transgenic males, wild-type non-transgenic females, transgenic Ag(PMB)1 males, and transgenic Ag(PMB)1 females. In addition, pupal mortality of females, transgenic and non-transgenic siblings, with X chromosome damages was assessed. Therefore, first-instar larvae were obtained by crossing 150 Ag(PMB)1 males × 150 G3 females in two small cages (17.5 × 17.5 × 17.5 cm), allotted into 20 trays containing 250 larvae each, and maintained until pupal development using our standard rearing protocol. All transgenic pupae were screened for fluorescence (3XP3DsRed marker, COPAS) and sexed. The number of transgenic and non-transgenic female pupae were counted (transgenic females cage 1 = 52; transgenic females cage 2 = 58; non-transgenic females cage 1 = 55; non-transgenic females cage 2 = 28). These female pupae with X chromosome damages were used to compute pupal mortality of females with X chromosome damages induced by the I-PpoI enzyme. Temperature and relative humidity were kept stable during all studies i.e. 27°C (± 1°C) and 75% relative humidity (± 10%).

Adult longevity in type 2 large cage.

We estimated the adult survival probability of male and female mosquitoes in seven indoor-large cages type 2 (5.0 × 1.22 × 2.60 m) for each strain, Ag(PMB)1 and wild-type, using progeny that resulted from crossing 150 Ag(PMB)1 females × 150 G3 males in small cages. We divided our adult longevity dataset into two blocks corresponding to two different times of data recording, three replicates for block 1 and four replicates for block 2. Daily, we removed and examined dead mosquitoes from type 2 large cages. Each of the type 2 large cages was equipped with a resting shelter, which corresponded to a humidity source, and three 500 mL cups containing a sugar source. Each cup was composed of 10% of sucrose solution, 0.1% methylparaben and approximately 1 mL acacia honey, with white absorbent paper placed on the top, allowing mosquitoes to land and get the sugar meal without drowning. Females were blood fed weekly with defibrinated and heparinized sterile cow blood using a Hemotek membrane feeder. As described by Facchinelli et al. (2015), type 2 large cages were arranged with swarming stimuli. In order to promote the swarming process of male mosquitoes in each cage, dawn lasted for 30 min from dark to full light, and full light lasted for 11h 30 min. Sunset lasted for 1 h 30 min, fading from the ceiling from full light to low light, overlapping with 60 min of twilight provided by horizon lights.

Results

No significant differences in adult survival between Ag(PMB)1 and wild-type siblings were detected in the type 2 cages (F= 5.08, df =1, P = 0.05), with no block effect between replicates (F= 0.33, df =1, P = 0.58). Our adult survival analysis indicated that cage size strongly affected the mean adult survival rate of wild-type siblings and Ag(PMB)1 mosquitoes with 50% mean mortality at 16 days (95% CI = 14-18 days) for wild-type and 19 days (95% CI 17-21 days) for Ag(PMB)1 in type 2 cages, substantially less the 28-30 days detected in small cages (17.5 x 17.5 x 17.5 cm) using

the same laboratory conditions (Facchinelli et al. 2019) (Table S1, Fig. S1). No statistical differences in male survival probability (F= 3.56, *df* =1, *P* = 0.09) with no block effect between replicates (F= 0.32, *df* =1, *P* = 0.58) were observed between Ag(PMB)1 and wild-type siblings in type 2 large cages (Table S1, Fig. 1). Ag(PMB)1 females did not differ from wild-type sibling females (F= 1.2, *df* =1, *P* = 0.27) with no variation between temporal replicates (F= 0.29 *df* =1, *P* =0.56). In agreement with Facchinelli et al. (2019), our data suggested that females are longer-lived than males in large cages. The time to mean 50% mortality in wild-type sibling females was estimated as 18 days (95% CI =15-21 days) and as 13 days for wild-type males (95% CI = 11-15 days). Similarly to the wild-type siblings, sex-specific difference in adult longevity of Ag(PMB)1 was observed in type 2 large cages, with Ag(PMB)1 showing 50% mortality at 20 days (95% CI 18-24 days) and 16 days (95% CI 13-19 days) for females and males, respectively.

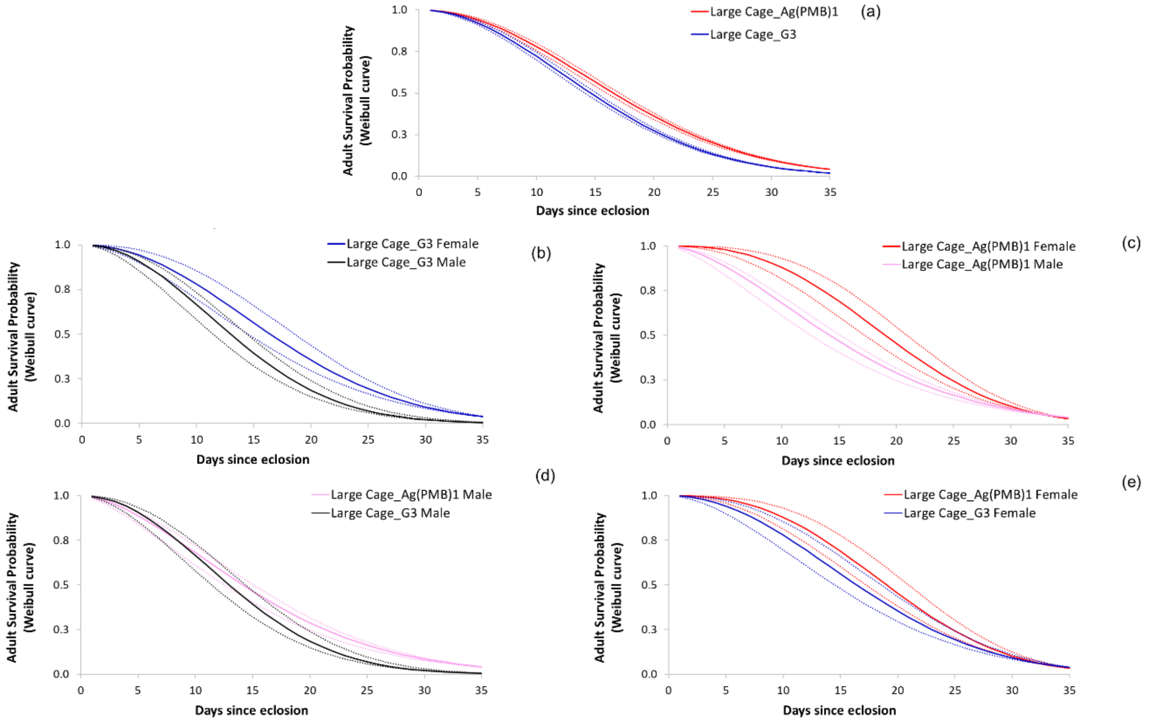

Figure S1. (a) Adult survival of wild-type and Ag(PMB)1 in indoor-large type 2 cages (5.0 × 1.22 × 2.60 m). Differences in adult longevity between male and female mosquitoes using (b) wild-type and (c) Ag(PMB)1 adults were displayed. Interpolated Weibull curve of adult survival using (d) male and (e) female of wild-type and Ag(PMB)1 and the respective lower and upper 95% confidence interval (dotted lines) were reported.

Table S1. Life history traits of the transgenic Ag(PMB)1 strain and its crosses with the wild-type G3 strain. For the Ag(PMB)1 transgenic strain, we report the average egg laying rate and hatching rate compared to the control (transgenic female × wild-type G3 male), the average larval mortality, pupal mortality and adult survival in small and type 2 cages compared to wild-type, the average percentage of transgenic offspring, and the percentage of males in the pooled progeny (± standard error).

**Life history traits**		**Data source**
Average number of eggs / female		
Ag(PMB)1 ♂ × G3 ♀	145.3 ± 11.8	Galizi et al., (2014)
Ag(PMB)1 ♀ × G3 ♂	199.9 ± 15.7	Galizi et al., (2014)
Normalized egg laying rate	75%	Galizi et al., (2014)
Hatching rate		
Ag(PMB)1 ♂ × G3 ♀	75.2% ± 7.6%	Galizi et al., (2014)
Ag(PMB)1 ♀ × G3 ♂	74.8% ± 8.5%	Galizi et al., (2014)
Normalized hatching rate	100.4%	Galizi et al., (2014)
Proportion of transgenic offspring		
Ag(PMB)1 ♂ × G3 ♀	48.9%	Galizi et al., (2014)
Ag(PMB)1 ♀ × G3 ♂	45.6%	Galizi et al., (2014)
Proportion of males in the pooled progeny		
Ag(PMB)1 ♂ × G3 ♀	94.6% ± 0.9%	Galizi et al., (2014)
Ag(PMB)1 ♀ × G3 ♂	50.5% ± 1.7%	Galizi et al., (2014)
Larval mortality rate		
Ag(PMB)1 ♂ × G3 ♀		
Ag(PMB)1	8.5 % ± 1.1 % [ns]	Facchinelli et al. (2019)
Wild-type non-transgenic siblings	8.5 % ± 1.1 %	Facchinelli et al. (2019)
Ag(PMB)1 ♀ × G3 ♂[a]		
Ag(PMB)1	10.1% ± 0.6% [ns]	Present study
Wild-type non-transgenic siblings	11.9% ± 1.3%	Present study
Pupal mortality rate		

Ag(PMB)1 ♂ × G3 ♀		
Total Ag(PMB)1	6.6 % ± 4.3 % [ns]	Facchinelli et al. (2019)
Total Wild-type non-transgenic siblings	6.6 % ± 4.3 %	Facchinelli et al. (2019)
Ag(PMB)1 ♂	6.6 % ± 4.3 % [ns]	Facchinelli et al. (2019)
Wild-type non-transgenic siblings ♂	6.6 % ± 4.3 %	Facchinelli et al. (2019)
Ag(PMB)1 ♀ with X chromosome-damage [b]	11.04% ± 1.98% *	Present study
Wild-type siblings ♀ with X chromosome-damage [b]	19.84 ± 1.62% *	Present study
Ag(PMB)1 ♀ × G3 ♂ [a]		
Total Ag(PMB)1	6.52% ± 0.55% [ns]	Present study
Total Wild-type non-transgenic siblings	8.20% ± 0.70%	Present study
Ag(PMB)1 ♂	6.74% ± 0.95% [ns]	Present study
Wild-type non-transgenic siblings ♂	9.04% ± 1.22%	Present study
Ag(PMB)1 ♀	6.5% ± 0.8% [ns]	Present study
Wild-type non-transgenic siblings ♀	7.4% ± 1.0%	Present study
Adult survival in small cages [c]		
Ag(PMB)1 ♂	28	Facchinelli et al. (2019)
Wild-type non-transgenic siblings ♂	28	Facchinelli et al. (2019)
Ag(PMB)1 ♀	30	Facchinelli et al. (2019)
Wild-type non-transgenic siblings ♀	30	Facchinelli et al. (2019)
Adult survival in large cages type 2[d]		
Total Ag(PMB)1	19 [17-21] ns	Present study
Total Wild-type non-transgenic siblings	16 [14-18]	Present study
Ag(PMB)1 ♂	16 [13-19] ns	Present study
Wild-type non-transgenic siblings ♂	13 [11-15]	Present study
Ag(PMB)1 ♀	20 [18-24] ns	Present study
Wild-type non-transgenic siblings ♀	18 [15-21]	Present study
Mating competitiveness (50% equals wildtype)		
Ag(PMB)1 *versus* Wild-type siblings	55.1% ± 8.2%	Facchinelli et al. (2019)

[a] Statistical difference between transgenic Ag(PMB)1 and wild-type non-transgenic individuals in terms of larval survival and pupal mortality were tested using a binomial generalized linear model: ns = not significant, * $P < 0.05$.

[b] Statistical difference between female pupal mortality of Ag(PMB)1 and Ag(PMB)1 with X chromosome-damage and between female pupal mortality of wild-type individuals and wild-type with X chromosome damage using a binomial generalized linear model: ns = not significant, * $P < 0.05$.

[c] 50% adult mortality on median days was reported. No significant differences in adult survival between transgenic Ag(PMB)1 and wild-type strains in small cages and no sex-specific differences have been observed in Facchinelli et al. (2019).

[d] Median adult survival time (number of days) calculated using the Kaplan–Meier method implemented in the survival package v2.37-4. For the median survival time, 95% confidence intervals were reported in brackets. Significant differences in 50% of adult survival between transgenic Ag(PMB)1 and wild-type strains were evaluated using two-way analysis of variance.: ns = not significant

References

Facchinelli, L., Valerio, L., Lees R.S., Oliva, C.F., Persampieri, T., Collins, C.M.,…Benedict M.Q. (2015). Stimulating *Anopheles gambiae* swarms in the laboratory: application for behavioural and fitness studies. Malaria Journal, 14, 271-280. doi:10.1186/s12936-015-0792-2.

Facchinelli, L., North, A.R., Collins, M.A., Menichelli, M., Persampieri, T., Bucci, A.,…Benedict, M.Q. (2019). Large-cage assessment of a transgenic sex-ratio distortion strain on populations of an African malaria vector. Parasites & Vectors 12, 70. doi:10.1186/s13071-019-3289-y.

Galizi, R., Doyle, L.A., Menichelli, M., Bernardini, F., Deredec, A., Burt, A.,…Crisanti A. (2014). Synthetic sex ratio distortion system for the control of the human malaria mosquito. Nature Communications, 5, 3977. doi:10.1038/ncomms4977.
