# Supplementary material for: Detecting the population dynamics of an autosomal sex ratio distorter transgene in malaria vector mosquitoes
Source: J Appl Ecol. 2020 Jul 20;57(10):2086–96. doi: 10.1111/1365-2664.13702 (PMC7594489; doi:10.1111/1365-2664.13702)
Supplement: Supplementary file 3 — Appendix S3 [file JPE-57-2086-s003.pdf]

Appendix S3. Description of the stochastic individual based simulation model of the overlapping generation cage experiments

The simulation model

The model assumes the population is comprised of juveniles, unmated adult females, adult males and mated adult females. Within these categories, the individuals are further differentiated by their age, their genotype with respect to the transgene (wildtype, Ag(PMB)1 hemi- or homozygous), and their X-chromosome type(s). We assume all X-chromosomes are a 'normal' type in the initial founding population, yet an 'affected' X-chromosome type is created whenever Ag(PMB)1 males produce X-bearing gametes. These X-chromosomes are thus the result of unsuccessful IPpoI expression and our model includes the possibility that they confer reduced fitness in females that carry them.

The model simulations are day-by-day iterations based on the following assumptions. Unmated females mate with a random male with probability m each day, which we set to $m = 0.9$ meaning that most females mate on the day of their emergence. On two days each week, each mated female has a probability P_{Lay} to lay a batch of eggs. The number of eggs in a batch is Poisson-distributed with expectation that depends on the genotype of both the female and her mate. The maximal expected egg batch size is θ, which we assume is achieved by wildtype females with normal X-chromosomes mated to wildtype males. This is reduced by a factor $1 - \psi$ if she carries an affected X-chromosome (or $(1 - \psi)^2$ if she carries two such X-chromosomes), and also by a factor $1 - \phi$ if her mate was transgenic. The parameters ψ and ϕ thus determine the extent to which the transgene might reduce female fertility. The egg genotypes are independently randomized using Mendelian probabilities, and the sex of each egg is male or female with equal probability unless the father has either one or two transgene copies, in which case the egg is male with probability b. In accordance with the experimental protocol, 500 eggs from each bi-weekly cohort are selected at

random to be reared to pupae. The development from egg to adult takes ten days (Facchinelli et al. 2010), and we assume that females with an affected X-chromosome survive pupation with probability $1 - \zeta$ (or $(1 - \zeta)^2$ for females with two affected X-chromosomes), with respect to all other individuals. The parameter ζ thus represents the cost of X-chromosome damage to successful pupation, and we set it to the value of 0.083 based on the measurements of pupal survival. After the ten days and after weighting by this factor, we re-randomise 200 individuals from the cohort and add them into the adult population.

The lifespan of each adult male and mated female is randomised from a Weibull-distribution whose shape (λ) and scale (k) parameters were fitted from the survival assays ($k_{Male} = 1.77$, $\lambda_{Male} = 16.32$, $k_{Female} = 2.21$, $\lambda_{Female} = 20.20$). The simulation was initiated by mirroring the population initiation phase and Ag(PMB)1 introduction shown in Fig. 1. The model was coded in C++ and compiled with the Intel compiler version 14.0.2.
