# Supplementary material for: Detecting the population dynamics of an autosomal sex ratio distorter transgene in malaria vector mosquitoes
Source: J Appl Ecol. 2020 Jul 20;57(10):2086–96. doi: 10.1111/1365-2664.13702 (PMC7594489; doi:10.1111/1365-2664.13702)
Supplement: Supplementary file 5 — Appendix S5 [file JPE-57-2086-s005.pdf]

Appendix S5. A mathematical description of parameter inference.

The Prior distribution

We used a prior distribution that was uniform with respect to all of the five parameters being estimated. The distribution bounds are the extremities of the parameter ranges shown in Fig. 3, specifically:

1. The cost to Ag(PMB)1 daughters fertility (ψ) ranged from 0 to 1.
2. The cost to Ag(PMB)1 male fertility (ϕ) ranged from 0 to 0.5.
3. Sex bias caused by IPpo1 expression (b) ranged from 0.7 to 1.
4. The egg laying probability (P_{Lay}) ranged from 0.1 to 1.
5. The maximal expected egg batch size (θ) ranged from 100 to 220.

The ABC distance measures

Our parameter inference was based on five measures of distance between a simulated data set and the empirical cage data. As described below, we used two distance measures from the transgene frequency time-series, two from the sex-ratio among transgenic pupae, and one from the egg number time-series.

Transgene frequency data

We denote by $\{a_d^i\}_{d=1}^{T}$ the time series from the ith cage ($i \in \{1,2,3\}$) of the frequency of Ag(PMB)1 among pupae, over all the days on which this was observed $d = 1..T$. We first smooth these series by transforming them into two-week moving average time-series, yielding three transformed series that we denote by $\{S(a_d^i)\}_{d=3}^{T-2}$. Note that the smoothed time series are four data points fewer than the original series because there are two data points per week in the original series. We next computed the sum of square differences between $\{a_d^i\}$ and $\{S(a_d^i)\}$ for each of the three series pairs, which we denote by $R(a^i)$. Specifically, $R(a^i) = \sum_{d=3}^{T-2}(a_d^i - S(a_d^i))$^2. We apply the same smoothing and residual functions to the

simulated data $\{b_d^j\}_{d=1}^T$ (where j refers to the jth simulation with the given parameters) to get corresponding variables $\{S(b^j)\}$ and $R(b^j)$. We can now calculate two distance measures between the observed and simulated data:

1. $d_1 = \sum_{i=1}^{3}\sum_{j=1}^{N_{sims}}[\sum_d \left(S(a_d^i) - S(b_d^j)\right)^2]$ is the sum of square differences between all corresponding pairs of smoothed empirical and smoothed simulated transgene frequency time-series.
2. $d_2 = \sum_{i=1}^{3}\sum_{j=1}^{N_{sims}}\left(R(a^i) - R(b^j)\right)^2$ is the sum of square differences between all corresponding pairs of real and simulated transgene frequency residuals.

Transgene sex-ratio data

In each cohort of pupae that were observed in the type 2 cages, the sex-ratio among transgenic pupae was recorded. Since we did not expect any temporal change in the transgenic sex-ratio, we computed the mean and variance of this data from cage i, μ_i and σ_i^2 for the three cages, $i \in \{1,2,3\}$. Similarly, we computed the mean and variance of this variable from the simulated data, ${\mu'}_j$ and $\sigma_j^{2\prime}$ for simulation j. This resulted in the next two distance measures:

3. $d_3 = \sum_{i=1}^{3}\sum_{j=1}^{N_{sims}}\left(\mu_i - {\mu'}_j\right)^2$ is the sum of square differences between all simulated and empirical mean sex ratio means.
4. $d_4 = \sum_{i=1}^{3}\sum_{j=1}^{N_{sims}}\left(\sigma_i^2 - \sigma_j^{2\prime}\right)^2$ is the sum of square differences between all simulated and empirical within cage variances in sex ratio.

Egg count data

Finally, the experiment recorded the number of eggs that were produced after each blood-feeding opportunity (twice per week), both in the type 1 cages (before Ag(PMB)1 mosquitoes were introduced), and in the type 2 cages. We denote by $\left\{\{e_d^{(1,i)}\}_{d=1}^{T_1}\right\}_{i=1}^{2}$ and $\left\{\{e_d^{(2,i)}\}_{d=1}^{T_2}\right\}_{i=1}^{3}$ the egg number time series from the two type 1 cages and three type 2 cages respectively. We smoothed these time series using the same 2-week moving average transformations that we

applied to the transgene frequency time-series, yielding $\left\{S(\{e_d^{(1,i)}\}_{d=3}^{T_1-2})\right\}_{i=1}^{2}$ and $\left\{S(\{e_d^{(2,i)}\}_{d=1}^{T_2})\right\}_{i=1}^{3}$. We similarly obtained corresponding smoothed egg number time-series from the simulations which we denote by e', in place of e. The final distance measure is the sum of square differences between smoothed and observed egg number time series, across cages and replications:

5. $d_5 = \sum_{i=1}^{2}\sum_{j=1}^{N_{sims}}[\sum_d \left(S\left(e_d^{(1,i)}\right) - S\left(e'^{(1,j)}_d\right)\right)^2] + \sum_{i=1}^{3}\sum_{j=1}^{N_{sims}}[\sum_d \left(S\left(e_d^{(2,i)}\right) - S\left(e'^{(2,j)}_d\right)\right)^2]$

Note that we do not construct a distance measure from the variance in egg number. This is because the model was unable to replicate the high variability that we observed, as discussed in the main text.

Monte-Carlo inference algorithm

We inferred the Posterior distribution (shown in Fig. 3) by iterating the following algorithm. First, we selected a parameter vector (a ‘particle’) at random from the prior distribution. We simulated the experiment using these parameters 20 times and calculated the distances $d_1 \dots d_5$ from the empirical data. We repeated this process 200,000 times to obtain a set of particles and associated distance vectors. We retained all particles for which all the five distances were in the lowest 0.3014 quantile for that measure. This yielded the 200 Posterior points shown in Fig. 3.
