# Supplementary material for: Detecting the population dynamics of an autosomal sex ratio distorter transgene in malaria vector mosquitoes
Source: J Appl Ecol. 2020 Jul 20;57(10):2086–96. doi: 10.1111/1365-2664.13702 (PMC7594489; doi:10.1111/1365-2664.13702)
Supplement: Supplementary file 6 — Appendix S6 [file JPE-57-2086-s006.pdf]

Appendix S6. Dynamics of three wild-type populations with overlapping generations after release of heterozygous transgenic Ag(PMB)1 males.

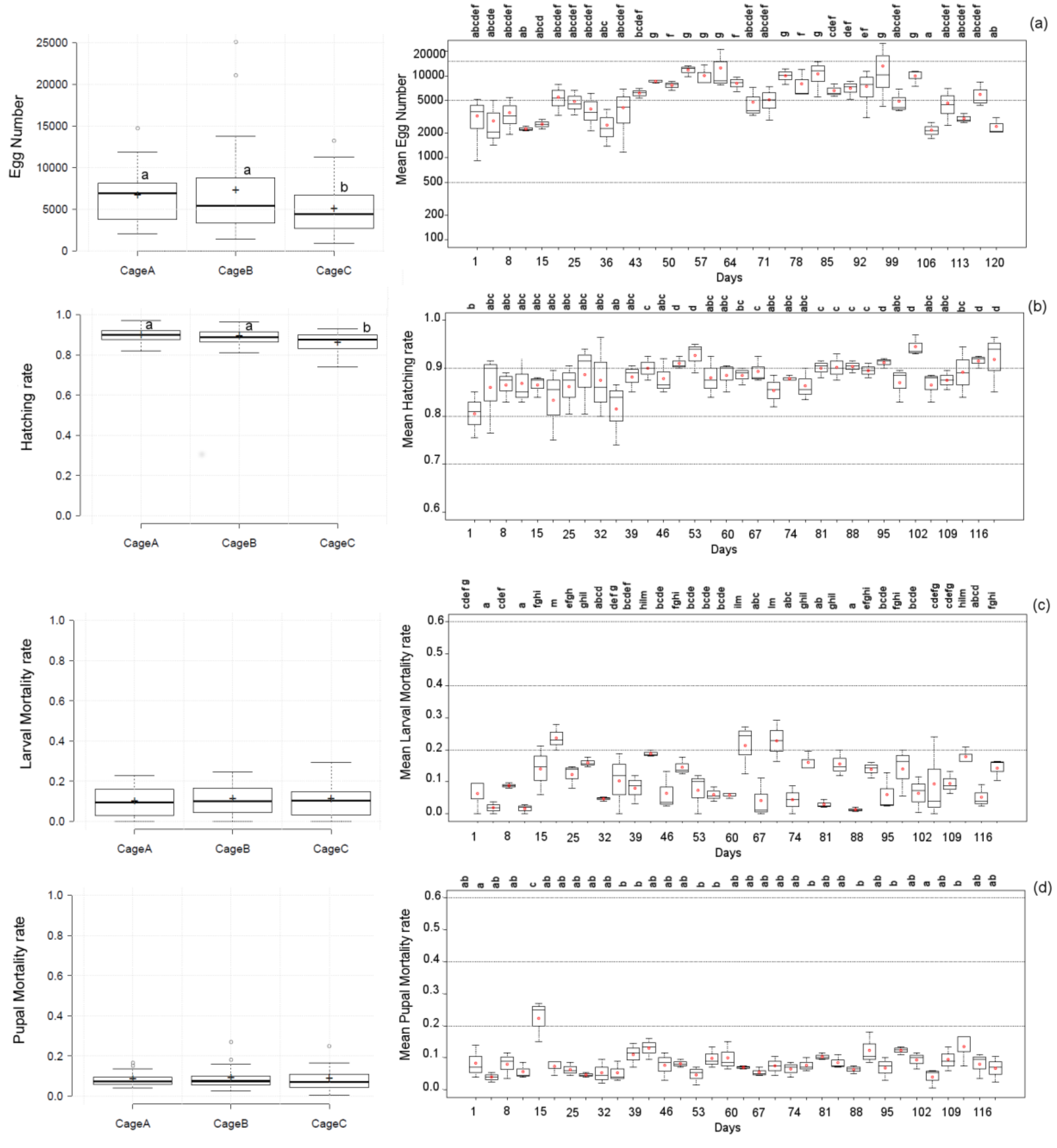

Fig. S1. Life-history parameters recorded over 120 days in three age-distributed, generation-overlapping G3 populations after seeding each large cage with 20% transgene frequency (type 2 cage A-C). Average and time-dependent (Days) (a) total number of eggs laid by females, (b) hatching rate, (c) larval mortality and (d) pupal mortality were shown. Mean values computed among cages (right side) and over the time (left side) showing the same letter (upper bar) were not significantly different at $P \leq 0.05$ according to the post-hoc Tukey's HSD test after providing significance within the generalized linear model (GLM).

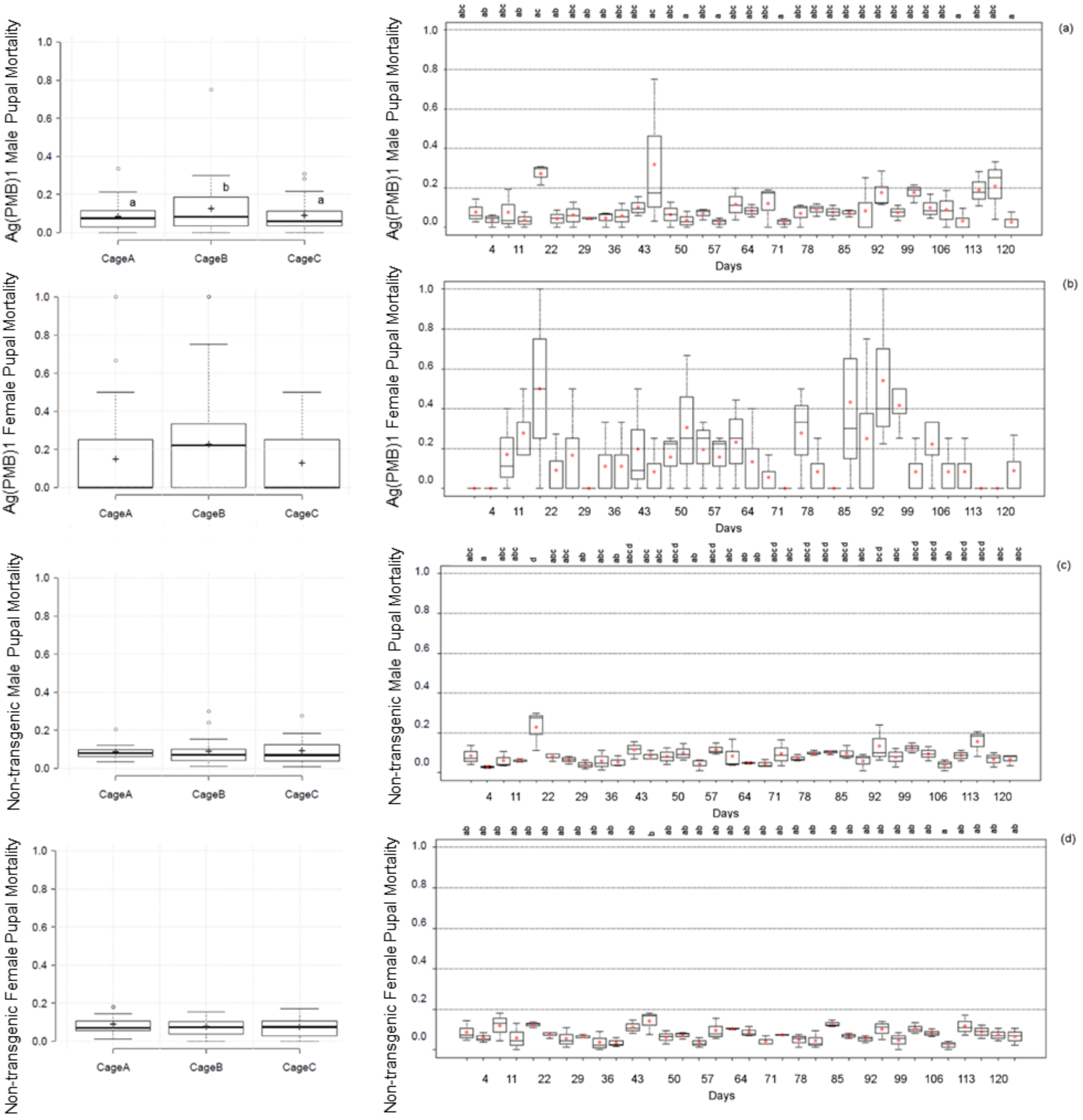

Fig. S2. Male and female pupal mortality recorded over 120 days in three age-distributed, generation-overlapping G3 populations after seeding each large cage with 20% transgene frequency (type 2 cage type 2 A-C). Average (right side) and time-dependent (left side) pupal mortality of (a) transgenic Ag(PMB)1 males, (b) transgenic Ag(PMB)1 females, (c) non-transgenic males, and (d) non-transgenic females were reported. Mean values showing the same letter (upper bar) were not significantly different at P ≤ 0.05 according to the post-hoc Tukey's HSD test after providing significance within the generalized linear model (GLM).

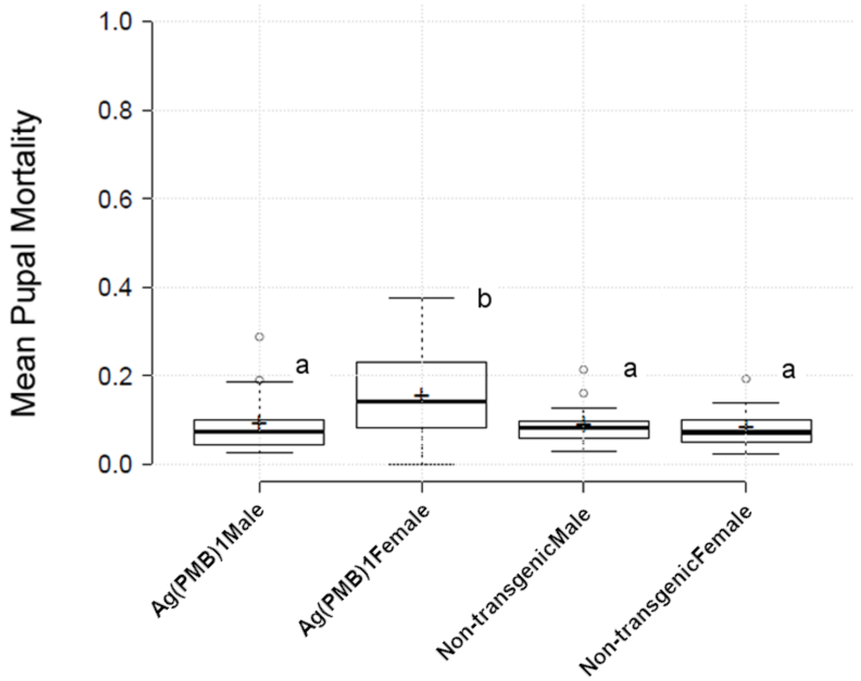

Fig. S3. Mean pupal mortality of Ag(PMB)1 males (0.086 ± 0.011), Ag(PMB)1 females (0.150 ± 0.019), non-transgenic males (0.084 ± 0.006) and non-transgenic females (0.076 ± 0.006) recorded over the course of 120 days in three age-distributed, generation-overlapping G3 populations after seeding each large cage with 20% transgene frequency. Mean values showing the same letter (upper bar) were not significantly different at $P \leq 0.05$ according to the post-hoc Tukey's HSD test after providing significance within the generalized linear model (Wald test=6.49, df=3, $P < 0.01$).
